# Conformation Transition of Intracellular Part of Glucagon Receptor in Complex With Agonist Glucagon by Conventional and Accelerated Molecular Dynamics Simulations

**DOI:** 10.3389/fchem.2019.00851

**Published:** 2019-12-17

**Authors:** Qifeng Bai, Shuoyan Tan, Horacio Pérez-Sánchez, Haixia Feng, Liya Feng, HuanXiang Liu, Xiaojun Yao

**Affiliations:** ^1^Key Lab of Preclinical Study for New Drugs of Gansu Province, Institute of Biochemistry and Molecular Biology, School of Basic Medical Sciences, Lanzhou University, Lanzhou, China; ^2^School of Pharmacy, Lanzhou University, Lanzhou, China; ^3^Computer Science Department, Universidad Católica San Antonio de Murcia (UCAM), Murcia, Spain

**Keywords:** G-protein-coupled receptors, glucagon receptor, molecular dynamics simulations, accelerated molecular dynamics simulations, conformation transition

## Abstract

The inactive conformations of glucagon receptor (GCGR) are widely reported by crystal structures that support the precision structure for drug discovery of type 2 diabetes. The previous study shows that the intracellular part is open in the glucagon-bound GCGR (glu-GCGR) and closed in the apo-GCGR by accelerated molecular dynamics (aMD) simulations. However, the crystal structure of GCGR in complex with partial agonist shows that the intracellular part is closed in the inactive conformation. To understand the differences between the studies of aMD simulations and crystal structure, the 2,500 ns conventional molecular dynamics (cMD) simulations are performed on the simulated model of glu-GCGR. The result shows that the transmembrane helices (TMH) 6 of glu-GCGR is outward ~4 Å to drive the intracellular part of glu-GCGR open until ~390 ns cMD simulations. The (TMH) 6 of glu-GCGR becomes closed after ~490 ns cMD simulations, which are consistent with the crystal structure of GCGR in complex with the partial agonist. To further elucidate the activation mechanism of GCGR deeply, the simulated models of glu-GCGR, apo-GCGR, and antagonist-bound GCGR (ant-GCGR) are constructed to perform 10 of parallel 300 ns aMD simulations, respectively. The results show that both of glu-GCGR and apo-GCGR can generate the open conformations of the intracellular part. But the glu-GCGR has the higher percentage of open conformations than apo-GCGR. The ant-GCGR is restricted to generate the open conformations of the intracellular part by antagonist MK-0893. It indicates that the glu-GCGR, apo-GCGR, and ant-GCGR can be distinguished by the aMD simulated method. Free energy landscape shows that the open conformations of the intracellular part of GCGR are in intermediate state. Our results show that aMD simulations enhance the space samplings of open conformations of GCGR via adding extra boost potential. It indicates that the aMD simulations are an effective way for drug discovery of GCGR.

## Introduction

The human glucagon receptor (GCGR), which belongs to one member of class B G-protein-coupled receptors (GPCRs), is a potential drug target of type 2 diabetes (Cho et al., 2012). The first crystal structure of the GCGR, which is resolved without any ligand, is lack of the binding site information for studying the interaction mechanism between a ligand and the GCGR (Siu et al., 2013). The GCGR in complex with antagonist MK-0893, which is determined by X-ray diffraction, supplies an accurate model for antagonist design (Jazayeri et al., 2016). With the research development of GCGR structures, the GCGR with crystal extracellular domain (ECD) is reported for understanding the signal transduction mechanism of class B GPCRs (Zhang H. et al., 2017). The structure of the GCGR in complex with glucagon analog is determined for elucidating a two-domain binding mechanism in which C-terminal and N-terminal bind to ECD and transmembrane domain (TMD) binding pocket, respectively (Zhang H. et al., 2018). The glucagon-like peptide-1 receptor (GLP-1R) and the GCGR that belonged to members of class B GPCRs have very similar structures, but they have different regulated mechanisms on blood sugar level. The GCGR decreases the blood sugar level through its inactive conformation. The inhibited binding position of GCGR antagonist is at the boundary between transmembrane helices (TMH) and lipid membrane. It can restrict the outward movement of TMH to reduce post-prandial and fasting glucose concentrations (Bagger et al., 2011). However, the GLP-1R decreases the blood sugar level through its active conformation. The binding pocket of active conformation of GLP-1R is surrounded by seven TMH (Zhang Y. et al., 2017).

The molecular dynamics (MD) simulations supply a reliable way to study the dynamical conformation transition of GCGR at the atomic level. It has been successfully used to study the dynamical conformation transition and binding mechanism between ligands and GPCRs (Li et al., 2010; Bai et al., 2014b; Bai and Yao, 2016), especially class A GPCRs (Feng et al., 2012; Bai et al., 2014a; Wang et al., 2018; Zhang X. et al., 2018). In the previous reports, the MD simulations indicate that the extracellular domain (ECD) of apo-GCGR can be in open or closed state for elucidating glucagon binding mechanism (Yang et al., 2015). The similar phenomena of closed or open ECD domain also happen in apo-GLP-1R, which is studied by MD simulations and the Markov state model (Zhang et al., 2019). These scientific researches give a computational model for studying the two-domain binding mechanism of class B GPCRs. Besides, the accelerated MD (aMD) simulations are performed on the apo-GCGR and the GCGR in complex with glucagon to study the activation mechanism of the GCGR. It indicates that the GCGR is in the active conformation when the glucagon binds to the pocket surrounded by 7TMH, and the apo-GCGR keeps the inactive state during aMD simulations (Li et al., 2016). This study does not elucidate the dynamical conformation characteristics between the GCGR and an antagonist, which can decrease the blood sugar level to treat type 2 diabetes mellitus. And it shows the different phenomenon with the crystal structure of the GCGR in complex with a partial agonist, which shows the inactive conformation of the intracellular part of the GCGR (Zhang H. et al., 2018).

To understand the differences between the crystal structure of the GCGR in complex with a partial agonist (Zhang H. et al., 2018) and the previous study of aMD simulations (Li et al., 2016), the 2,500 ns of conventional molecular dynamics (cMD) are performed on the same model of glucagon-bound GCGR (glu-GCGR) as previously reported (Li et al., 2016). The results of cMD simulations show the similar structure of the intracellular part of the GCGR with the crystal structure of the GCGR in complex with a partial agonist. Besides, 10 of parallel 300 ns aMD simulations are carried out on glu-GCGR, apo-GCGR, and antagonist-bound GCGR (ant-GCGR), respectively. It points out the differences of structural characteristics of glu-GCGR, apo-GCGR, and ant-GCGR by statistics of aMD simulated trajectories. Free energy landscape profiles the energy change during the conformational transition process of the intracellular part of the GCGR. Our studies give the deep insight into the activation mechanism of the GCGR by cMD and aMD simulations.

## Materials and Methods

### Model Preparation

To repeat previous literature experiments, the simulated model of glucagon-bound GCGR (glu-GCGR) is extracted from the previous report of aMD simulations (Li et al., 2016). The antagonist-bound GCGR (ant-GCGR) is obtained from PDB database (PDB ID: 5EE7) (Jazayeri et al., 2016). The miss residues and disulfide bond are automatically dealt with by Schrödinger software release 2015 (Sastry et al., 2013). The apo-GCGR system is built by removing the antagonist MK-0893 of the ant-GCGR (PDB ID: 5EE7). The ant-GCGR and apo-GCGR are constructed with the solvation-membrane box dimensions of 85 × 85 × 68 Å^3^. The glu-GCGR is immersed in the solvation-membrane box with the dimensions of 85 × 85 × 115 Å^3^. The TMH of the GCGR are placed along the z axis and are orthogonal to the 1-palmitoyl-2-oleoyl-sn-glycero-3-phosphocholine (POPC) lipids of which size is 85 Å × 85 Å. The TIP3P model (Jorgensen et al., 1983) is chosen for water box and 0.15 M NaCl is used to neutralize the computational model systems. The force field parameters of antagonist MK-0893 are generated by the paramchem server of CHARMM General Force Field (CGenFF) (Vanommeslaeghe et al., 2012).

### Conventional and Accelerated Molecular Dynamics Simulations

The simulated model of glu-GCGR except for the membrane is fixed to perform the 50 ps of minimization and 500 ps of equilibrium simulations. Then, only the ligands and protein are constrained for running 100 ps minimization and 500 ps cMD simulations. In the next step, the total 20 ns of equilibrating cMD simulations is performed on the full released systems. At last, 2,500 ns of production cMD simulations are run on glu-GCGR. All cMD simulations are carried out under the constant temperature of 310 K and constant pressure of 1 bar.

The NAMD (Phillips et al., 2005) soft package (version 2.11) is used to run the cMD simulations on the basis of CHARMM 27 force field (Mackerell et al., 1998) in the infinitely periodical water-lipid box. The energy minimizations employ the conjugate gradient method in the NAMD software. The non-bonded cutoff of 12 Å is chosen to calculate the electrostatic interaction based on the particle-mesh Ewald (PME) method (Darden et al., 1993). The Langevin barostat and Langevin thermostat are used to control constant temperature and pressure of simulated system of the glu-GCGR (Feller et al., 1995). The simulated step is set to 2f for the simulated system of the glu-GCGR. And the frames of cMD simulations are stored every 10 ps.

The accelerated MD (aMD) simulations have been successfully applied into the studies of protein conformational transition (Miao et al., 2013; Bai et al., 2014c). It can sample the molecular configuration space efficiently via adding boost potential Δ*V*(*r*). The modified potential ΔV^*^(*r*) is equal to the sum of original potential *V*(*r*) and boost potential Δ*V*(*r*). The modified potential ΔV^*^(*r*) and boost potential Δ*V*(*r*) are shown in Equation (1):

(1) $$\begin{array}{l} \;V^{*}\left(r\right)=V\left(r\right)+\Delta V\left(r\right)\\ \Delta V\left(r\right)=\{\begin{array}{cc} 0, & V\left(r\right)\geq E\\ \frac{{\left(E-V\left(r\right)\right)}^{2}}{\alpha +E-V\left(r\right)}, & \;V\left(r\right)<E \end{array} \end{array}$$

where *E* is the threshold energy, and α is the acceleration factor. The 10 of parallel dual-boost aMD simulations run 300 ns on the simulated systems of glu-GCGR, apo-GCGR, and ant-GCGR after 20 ns equilibrating cMD simulations. The values of threshold energy and acceleration factor are calculated (Miao et al., 2013) as follows (see Equation 2):

(2) $$\begin{array}{l} {\text{ E}}_{\text{total}}={\text{V}}_{\text{total\_avg}}+0.16^{*}{\text{N}}_{\text{atoms}}\\ \;\alpha_{\text{total}}=0.16^{*}{\text{N}}_{\text{atoms}}\\ {\text{E}}_{\text{dihed}}={\text{V}}_{\text{dihed\_avg}}+0.3^{*}{\text{V}}_{\text{dihed\_avg}}\\ \alpha_{\text{dihed}}=0.3^{*}{\text{V}}_{\text{dihed\_avg}}/5 \end{array}$$

in which *N*_atom_ is the total atom number of the entire system. The values of *V*_total_avg_ and *V*_dihed_avg_ are obtained from the average total potential energy and the average dihedral energy in cMD simulation, respectively. Total 9,000 ns aMD simulations are performed on the simulated systems of glu-GCGR, apo-GCGR, and ant-GCGR. And the trajectory snapshots are saved every 10 ps for each system. The residues L253 and Y343 are chosen to analyze the open and closed states of glu-GCGR, apo-GCGR, and ant-GCGR.

### Free Energy Landscape and Weak Interaction Analysis

The free energy landscape can show the energy change during the conformational transition process of the intracellular part of the GCGR. The free energy landscape is calculated as shown in Equation (3):

(3) $$\begin{array}{l} \Delta G=-k_{B}T\ln\;g\left(x,y\right) \end{array}$$

where *k*_B_ and *T* represent the Boltzmann constant and the temperature, respectively. x and y represent the two principal components that are the distance between the Cα atoms of L253 and Y343 of the GCGR and the root mean square deviation (RMSD) of the GCGR, respectively.

The graphical topological analysis named independent gradient model (IGM) (Lefebvre et al., 2017) is used to produce the interactive isosurfaces between L253 and Y343 of the GCGR. The weak interaction is computed by Equation (4):

(4) $$\begin{array}{l} {\delta \text{g}}^{\text{inter}}=|\nabla \rho^{\text{IGM},\text{inter}}|-|\nabla \rho^{\text{inter}}| \end{array}$$

in which ∇ρ^IGM, inter^ is the sum of absolute values of every atomic electron density of the intermolecular fragment. ∇ρ^inter^ is the sum of every atomic electron density of the intermolecular fragment. Here, the Multiwfn software (Lu and Chen, 2012) is used to calculate the IGM. The VMD software (Humphrey et al., 1996) is employed to draw the isosurfaces between residues L253 and Y343 based on the calculated results of Multiwfn. About 91,000 grids are generated to profile isosurfaces between residues L253 and Y343. The isosurfaces are generally divided as follows: blue represented attractive interaction such as hydrogen bonding, red represented repulsive interaction such as molecular steric effect, and green represented van der Waals interactions.

## Results and Discussions

### The Conformational Change of GCGR During 2,500 ns cMD Simulations

**Figure 1:**
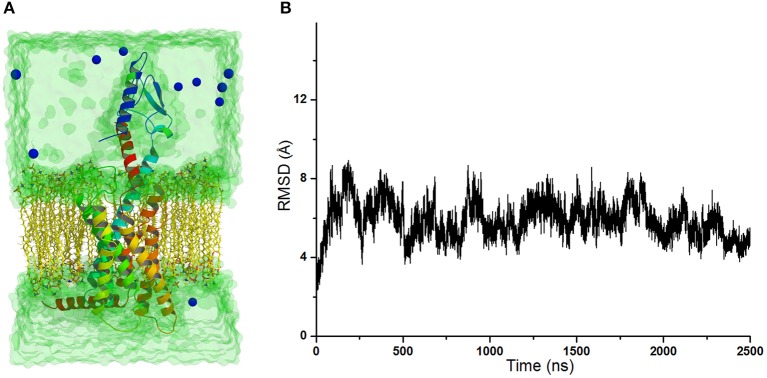
**(A)** The simulated model of the GCGR in complex with water, ions, glucagon, and lipids. To show the GCGR model clearly, part of water, ions, and lipids are hidden. **(B)** RMSD of backbone atoms of glu-GCGR vs. 2,500 ns cMD simulations.

The crystal structure of GCGR in complex with the partial agonist peptide indicates that the intracellular part keeps the inactive conformation (Zhang H. et al., 2018). However, the results of accelerated molecular dynamics (aMD) simulations show that the active conformations are predominant in the intracellular parts of the GCGR in complex with agonist glucagon (Li et al., 2016). To understand these different results, the simulated model of the GCGR is built in complex with glucagon, ions, water, and lipids (see Figure 1A). The total 2,500 ns of production cMD simulations are carried out on this simulated system. The RMSD of backbone atoms of glu-GCGR keeps the relative equilibrium phase during 2,500 ns cMD simulations (see Figure 1B). The distance between the Cα atoms of residues L253 and Y343 is chosen as a key principal component to mark the active or inactive state (Li et al., 2016). In our experiment, we also measure the distance between the Cα atoms of residues L253 and Y343 based on 2,500 ns cMD simulated trajectories.

**Figure 2:**
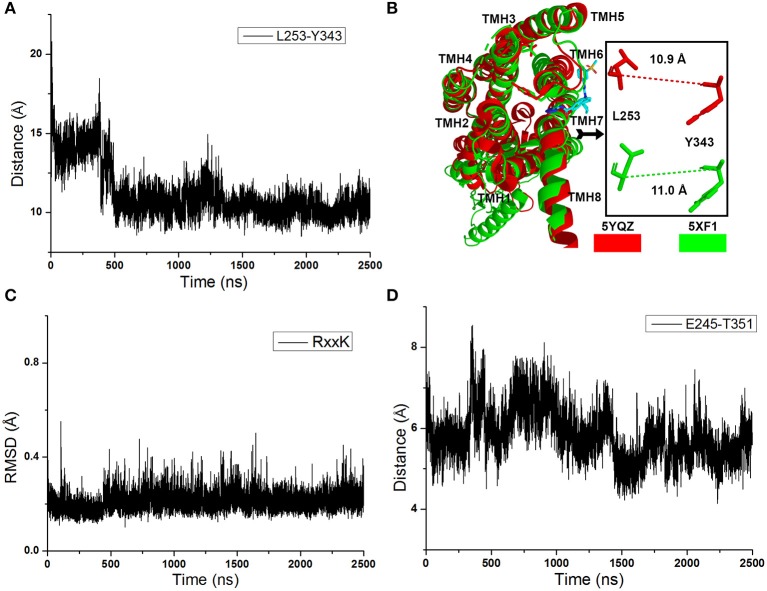
**(A)** The distance between the Cα atoms of L253 and Y343 of the glu-GCGR during 2,500 ns cMD simulations. **(B)** Alignment between the GCGR in the complex partial agonist (5YQZ) and the apo-GCGR with inactive conformation (5XF1). **(C)** RMSD of backbone atoms of the RxxK motif. **(D)** The distance between the carboxyl oxygen of E245 and the hydroxyl oxygen of T351 during 2,500 ns cMD simulations.

As shown in Figure 2A, the distance between the Cα atoms of L253 and Y343 increases to ~15 Å quickly at the initial cMD simulations. It means the TMH 6 of glu-GCGR is outward ~4 Å to drive the intracellular conformation of the glu-GCGR open. The results are consistent with the reported results (Li et al., 2016). Then, the distance between the Cα atoms of L253 and Y343 is changed until ~390 ns. The distance between the Cα atoms of L253 and Y343 becomes ~11 Å from ~490 ns MD simulations. The inactive crystal conformation of the GCGR shows that the distance is about 11 Å between the Cα atoms of L253 and Y343 (see Figure 2B). And the distance between the Cα atoms of L253 and Y343 is 10.9 Å in the crystal partial agonist-bound GCGR. The alignment shows that the partial agonist-bound GCGR has a similar intracellular structure with the inactive conformation of apo-GCGR (see Figure 2B). It indicates the intracellular helices of the GCGR trend to be inactive conformations whether or not the partial agonist binds to the GCGR. Our cMD simulations show similar results with the crystal structure of the partial agonist-bound GCGR.

**Figure 3:**
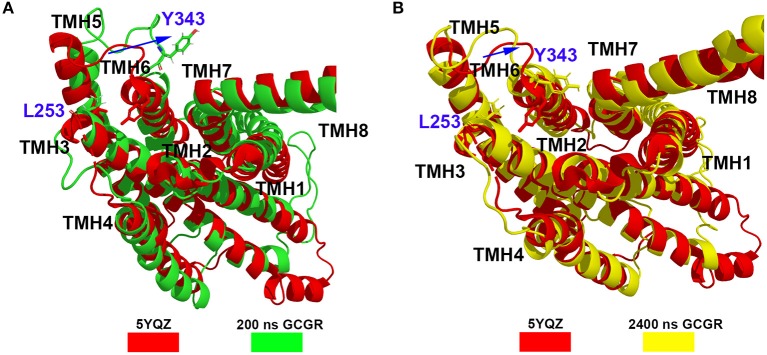
**(A)** Alignment between the GCGR in the complex partial agonist (5YQZ) and the 200th ns simulated structure of the GCGR. **(B)** Alignment between the GCGR in the complex partial agonist (5YQZ) and the 2,400th ns simulated structure of the GCGR.

Besides, the RxxK motif, which is in the end of TMH6, plays an important role in the activation of class B GPCRs. The RMSD of backbone atoms of the GCGR RxxK motif also shows a change in ~490 ns MD simulation (see Figure 2C). It indicates that the TMH6 changes its conformation. The crystal structure of the GCGR shows that the carboxyl of E245 contains two oxygen atoms, which are 4.3 and 5.9 Å away from the hydroxyl oxygen of T351, respectively. Due to the rotation of oxygen atoms of carboxyl of E245 in the cMD simulated process, the distance has a fluctuation between the carboxyl oxygen of E245 and hydroxyl oxygen of T351 during 2,500 ns cMD simulations (see Figure 2D). The 200th ns conformation, which is in the range of open intracellular helices, and the 2,400th ns conformation, which is in the range of closed intracellular helices, are extracted for comparing with the crystal GCGR (PDB ID: 5YQZ) in complex with the partial agonist (see Figures 3A,B). It shows that the intracellular part of TMH6 of the 200th ns GCGR, which contains the Y343, is away from the crystal structure of the GCGR (Figure 3A). But the 2,400th ns GCGR has a similar distance between Cα atoms of L253 and Y343 with the crystal GCGR. It is only a slight change in the intracellular part of TMH6 by comparing with the crystal GCGR (see Figure 3B). These results are consistent with a previous research report (Zhang H. et al., 2018).

### Conformational Change of Intracellular Helices by aMD Simulations

Li et al. (2016) show that the active conformations of glu-GCGR are preponderant during 220 ns aMD simulations. However, our cMD simulated results and the crystal structure of the GCGR in complex with a partial agonist indicate that the intracellular part of the glu-GCGR is inclined to keep the inactive conformation. These different results are attributed to the aMD simulations. The aMD simulations can reduce the energy barriers to enhance the conformational space sampling. So it infers that the apo-GCGR can also produce the active conformations by aMD simulations if enough aMD simulations are carried out. In this study, the simulated models of the glu-GCGR, apo-GCGR, and ant-GCGR are performed to verify our inference with 10 of parallel 300 ns aMD simulations, respectively.

**Figure 4:**
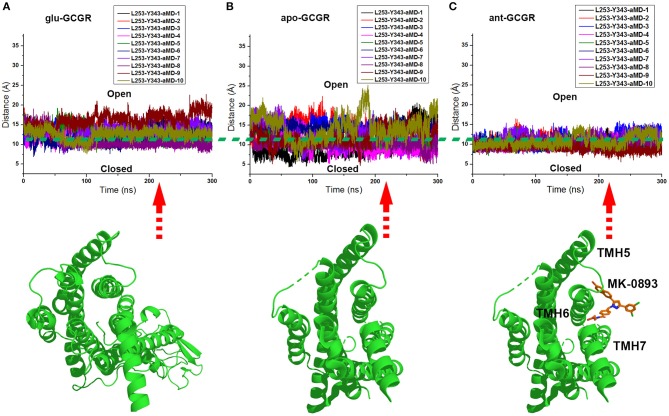
**(A)** The distance between the Cα atoms of L253 and Y343 of the glu-GCGR during 10 of parallel 300 ns aMD simulations. **(B)** The distance between the Cα atoms of L253 and Y343 of the apo-GCGR during 10 of parallel 300 ns aMD simulations. **(C)** The distance between the Cα atoms of L253 and Y343 of the ant-GCGR during 10 of parallel 300 ns aMD simulations.

The x and y axes choose the same range of values for plotting the distance between the Cα atoms of L253 and Y343 of the glu-GCGR, apo-GCGR, and ant-GCGR vs. 300 ns aMD simulations. The horizontal dotted line marks about 11 Å, which crosses over the same position of plotted contours of the glu-GCGR, apo-GCGR, and ant-GCGR (see Figures 4A–C). As shown in Figures 4A,B, some distances between the Cα atoms of L253 and Y343 of glu-GCGR and apo-GCGR reach more than 15 Å during 300 ns aMD simulations. These results are different from a previous report that shows the distance between the Cα atoms of L253 and Y343 of the apo-GCGR remains around 11 Å during aMD simulations (Li et al., 2016). The main reason is that the aMD simulations are the enhanced stochastic sampling process. Enough number of parallel MD simulations can get more small probability conformations of GCGR. Actually, some of aMD simulations of the apo-GCGR show that the distance between the Cα atoms of L253 and Y343 is around 15 Å during 300 ns aMD simulations (see Figure 4B). However, it is obvious that the area under the horizontal dotted line in the plotted contour of the glu-GCGR is less than in the plotted contour of the apo-GCGR. Due to the same parameters and the number of aMD simulations, it can infer that the glu-GCGR can generate more active conformations than the apo-GCGR during 300 ns aMD simulations. It indicates that the agonist glucagon can enhance the probability of GCGR active conformations. By compared with the cMD simulated results of the GCGR in complex with an agonist, the aMD simulations show the higher conformational thermodynamic energies of the GCGR in complex with an agonist (see Figure S1). Because the antagonist MK-0893 binds to the clefts of TMH5-TMH6 and TMH6-TMH7 (see Figure 4C), the distance between the Cα atoms of L253 and Y343 of the ant-GCGR is constrained to reach the 15 Å during 300 ns aMD simulations. Most of conformations of the GCGR in complex with the antagonist MK-0893 are kept in the inactive state during 10 of parallel 300 ns aMD simulations.

### Free Energy Landscape Surface Analysis

**Figure 5:**
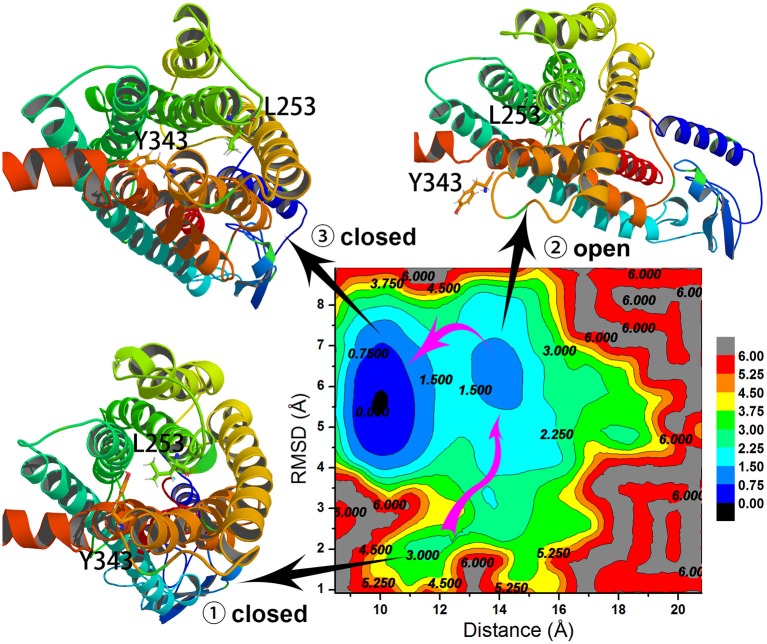
Free energy landscape profiled by the distance between the Cα atoms of L253 and Y343 of the GCGR and the RMSD of the GCGR.

**Figure 6:**
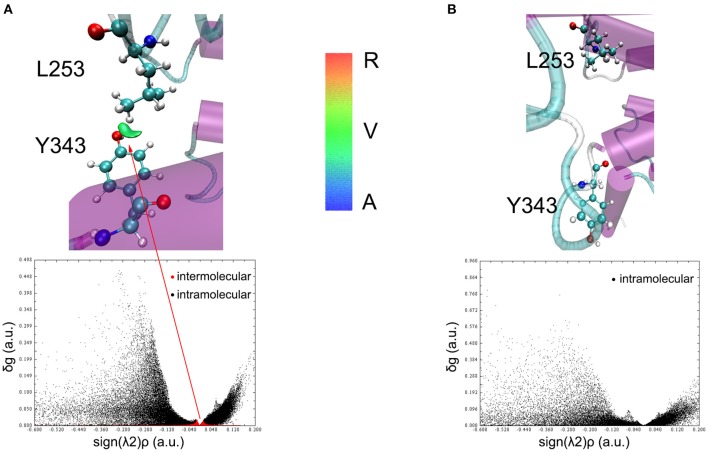
The weak interaction analysis between L253 and Y343. **(A)** Gradient isosurfaces between L253 and Y343 in the first simulated step. **(B)** Gradient isosurfaces between L253 and Y343 in the 2,400th ns simulated step. The color bar shows the blue, green, and red scale, which range from −0.04 to 0.02 au. Blue scale (A in color bar) represents the strong attractive interaction such as hydrogen bonding. Red scale (R in color bar) represents repulsive interaction such as molecular steric effect. Green scale (V in color bar) indicates van der Waals interactions.

To understand the closed and open process of the GCGR deeply, the underlying free energy landscape is profiled based on the simulated trajectories. The free energy landscape is drawn by two principal components, which are the distance between the Cα atoms of L253 and Y343 of the GCGR and the RMSD of the GCGR, respectively (see Figure 5). To elucidate the function of L253 and Y343 in the closed and open process of the GCGR during MD simulations, the first simulated step frame and the 2,400th ns simulated frame are chosen to calculate the weak interaction between L253 and Y343 of the GCGR, respectively (see Figures 6A,B). As shown in Figure 5, in the first simulated step, the closed conformation of the GCGR has a high energy barrier of ~3.0 kcal mol^−1^ on account of agonist glucagon in the pocket of the GCGR. In this state, the interaction between the residues L253 and Y343 is mainly van der Waals, which is the weak interaction to lock the residues L253 and Y343 of the GCGR (see Figure 6A). Then, the conformation of the GCGR opens quickly, which corresponds to ~1.5 kcal mol^−1^ energy barrier. In this state, the residues L253 and Y343 of the GCGR are separated from each other. And the van der Waals interaction disappeared between residues L253 and Y343 of the GCGR (see Figure 6B). The free energy landscape shows that the open conformation of the GCGR is in the intermediate state. The main reason is that the open intracellular part of the GCGR is not stable and with relative high energy barrier of ~1.5 kcal mol^−1^. Finally, the intracellular conformation of the GCGR falls into the lowest deep energy well (see Figure 5). The TMH3 and TMH6 of the GCGR trend to be closed, which correspond to the crystal structure of the GCGR in complex with a partial agonist (Zhang H. et al., 2018).

So far, the differences can be elucidated between the crystal structure of the GCGR in complex with a partial agonist and the studies of aMD simulations of Li et al. The aMD simulations add boost potential Δ*V*(*r*) on the original potential *V*(*r*) (see Equation 1). If the extra energy adds to the lowest deep energy well of Figure 5, the lowest deep energy well will become the higher deep energy well, which corresponds to the open conformational state of the GCGR. So the aMD simulations can enhance the space samplings of open conformations of the GCGR. In total, if we want to study the drug potency on the targets, an enough number of parallel aMD simulations should be performed on the apo-structure and ligand-bound models. The binding ability of ligands can be reflected by the statistics of pointed features based on the aMD simulated trajectories. The aMD simulations supply a reliable, fast, and precise way for drug discovery.

## Conclusions

In this study, the 2,500 ns of cMD and 9,000 ns of aMD simulations are performed to elucidate the differences between the crystal structure of the GCGR in complex with a partial agonist and the previous study of aMD simulations (Li et al., 2016). Our results profile the activation mechanism of the GCGR deeply based on a previous study of aMD simulations. It indicates that an enough number of parallel aMD simulations can distinguish the structural characteristics of the apo-GCGR, agonist- and antagonist-bound GCGR. The aMD simulations need less computational time to reach the similar results of cMD simulations. The aMD simulations not only can be used to study the interaction mechanism between the GCGR and ligands, but also can be further used to estimate the drug potency in other receptor targets.

## Data Availability Statement

All datasets generated for this study are included in the article/Supplementary Material.

## Author Contributions

QB, ST, HP-S, HF, HL, and XY design the entire experiments. QB, ST, HP-S, HF, and LF performed and analyzed cMD and aMD simulations. The manuscript is prepared with discussions and improvements from all authors.

### Conflict of Interest

The authors declare that the research was conducted in the absence of any commercial or financial relationships that could be construed as a potential conflict of interest.
