# Supplementary material for: Conformation Transition of Intracellular Part of Glucagon Receptor in Complex With Agonist Glucagon by Conventional and Accelerated Molecular Dynamics Simulations
Source: Front Chem. 2019 Dec 17;7:851. doi: 10.3389/fchem.2019.00851 (PMC6928006; doi:10.3389/fchem.2019.00851)
Supplement: Supplementary file 1 [file Data_Sheet_1.pdf]

Supplementary material

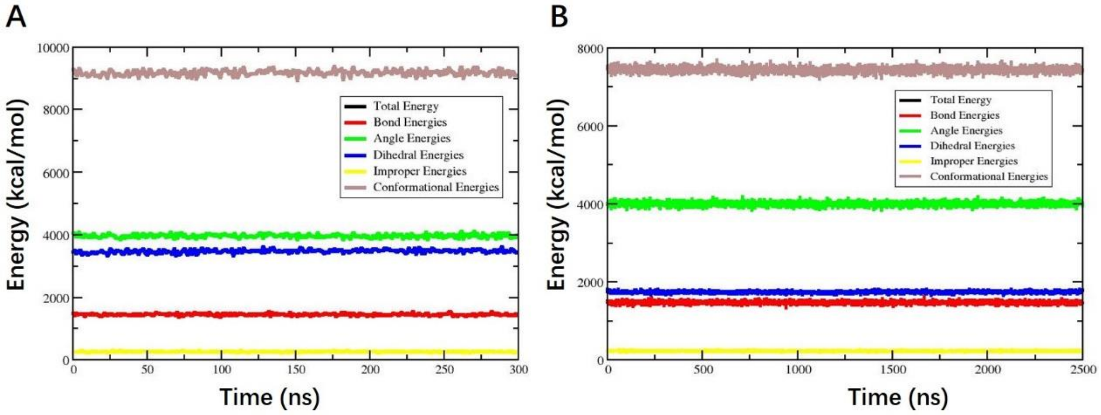

Figure S1. (A) The thermodynamic energy including bonding energies, angle energies, dihedral energies, improper energies and conformational energies versus 300 ns aMD simulations. (B) The thermodynamic energy including bonding energies, angle energies, dihedral energies, improper energies and conformational energies versus 2500 ns cMD simulations. The total energy is not shown in VMD GUI.
